# Stress corrosion cracking under extreme near-neutral GCC conditions, parametric and comparative study using phase field modeling

**DOI:** 10.1016/j.heliyon.2023.e18544

**Published:** 2023-07-21

**Authors:** Abdullah Alsit, Hasan Hamdan, Aghyad B. Al Tahhan, Mohammad Alkhedher

**Affiliations:** a Mechanical Engineering Department, College of Engineering, Abu Dhabi University, Abu Dhabi 59911, United Arab Emirates

**Keywords:** Petroleum sector, Oil and gas pipelines, X70 steel, Phase field modeling, Stress corrosion cracking, Film rupture dissolution repassivation mechanism, And corrosion rates

## Abstract

Stress Corrosion Cracking (SCC) is a failure mechanism that occurs when certain materials are subjected to both external or residual stresses and corrosion. This combined effect leads to the development of cracks in the susceptible materials. Submerged steel pipelines in the petroleum sector are built of low-alloy steels having a ferrite-cementite composition, including API 5L X70. Such materials are sensitive to SCC damage in aqueous systems. The film rupture dissolution repassivation (FRDR) process is used in this study to evaluate the cracks and pits growth in oil and gas pipelines in the Gulf area under diverse SCC environmental conditions. The SCC crack propagation and pit growth under near-neutral environmental conditions were analyzed using phase field modelling. X70 steel under NS4 the solution was used for the analysis to represent the anodic dissolution film rupture mechanism. A parametric study was done to study the impact of different electrochemistry and phase field parameters on crack growth behaviour. The study assess the susceptibility to SCC caused by an pit by employing diverse settings to evaluate the impact of corrosion parameters and the interaction among the FRDR mechanism. The corrosion rates are influenced by the interface kinetics coefficient (L), which exhibits an accelerated effect as L increases. This transition from fracture-controlled to dissolution-controlled SCC growth occurs until the system reaches the diffusion limit, beyond which further increases in L do not significantly impact corrosion rates. Moreover, higher values of the kinetic coefficient (k) advance the creation of SCC cracks at the crack front, resulting from corrosion originating from pitting at the crack mouth. This process leads to the refinement of the pit and its transformation into a crack. A comparison analysis was utilized to validate our simulation under a near-neutral NS4 solution for X70 steel by correlating the findings with other numerical methods for crack growth utilizing the same material and environmental parameters. The results show decent agreement with the comparative study.

## 1 Introduction

Stress corrosion cracking (SCC) is a type of corrosion that appears when a materials are exposed to both applied load and corrosive conditions [1]. It is a common problem in a variety of industries, including oil and gas, nuclear power, and aerospace. SCC occurs when the material is under tensile stress, which means that it is being pulled or stretched [2]. This can happen when a material is subjected to mechanical forces, such as during welding or bending. At the same time, the material must be exposed to a corrosive environment, such as water or certain chemicals. The combination of these factors can weaken the material and make it more susceptible to cracking [3]. SCC can also occur at low-stress levels, making it difficult to detect and prevent. Several factors can contribute to SCC, these include the type and concentration of the corrosive environment, the temperature and humidity of the surrounding, the type of material, and the presence of other aspects that can affect the material's susceptibility to corrosion, such as metallurgical defects or stress concentrations [4]. One of these occurrences is SCC, which is likely the most prevalent but difficult engineering fracture and failure mechanism [5]. Environmentally aided cracking (EAC) is a catch-all name for the many processes underlying this occurrence. It is broadly categorized into three types: SCC, corrosion fatigue (CF), and hydrogen-induced cracking (HIC) are all examples of SCC [6]. A susceptible material, corrosive conditions, and adequate tensile stress must all occur concurrently for the occurrence of the SCC. Owing to elevated relative humidity, elevated ambient temperature, and sea salt pollution of the atmosphere, Arabian Gulf atmospheric conditions are extremely corrosive to most metallic constructions [7]. The Arabian Gulf's ambient environments are a major cause of air corrosion on metallic materials and coating deterioration [8,9]. There have been several mechanistic explanations proposed, and methods can be divided into two types. The first relates to crack growth models and processes in which the anodic corrosion interference at the pit mouth drives crack growth, such as the active path dissolution Mechanism [10] or approaches that rely on the film rupture/dissolution and repassivation (FRDR) method [11]*.* The following group comprises cathodic-driven crack development theories related to hydrogen infiltration and transport, including the hydrogen-enhanced decohesion (HEDE) mechanism [12], adsorption-induced dislocation emission (AIDE) [13] or hydrogen-enhanced localized plasticity (HELP) [14]*.* The characteristics of SCC can exhibit significant variations among different materials and even within the same material across different environments. This variability poses challenges in comprehensively understanding the numerous mathematical models proposed to explain SCC. Furthermore, it is crucial to acknowledge that many of these processes are not commonly exclusive and have the potential to transpire concurrently [15].

The SCC approach is typically classified into three phases [16]: crack initiation, stable crack growth, and ultimate failure, with each stage controlled by the same or distinct processes. The creation of a microscopic crack at a confined corrosion or mechanical flaw location is connected with pitting, intergranular damage, scrapes, weld faults, or design notches [17]. To obtain the dynamic boundary condition of a corrosive environment, numerical approaches same as the extended Finite Element Method (XFEM) [18], Arbitrary Lagrangian–Eulerian (ALE) methods [19], peridynamics [20], and cohesive zone modelling (CZM) [21] have been established. The phase field modeling (PFM) approach provides an alternative method for simulating dynamic boundaries and interface phenomena, offering a redefined perspective on these aspects. A differential equation model is used to adjust the boundary conditions at a junction in the phase field paradigm in order to produce a supplementary phase field parameter (*&varphi*) [22]. This phase field parameter has multiple separate quantities in every phase, with a seamless transition between the two quantities in a dispersed area nearby the interface. Integrating a set of partial differential equations that encompass the entire structure, it becomes possible to address the issue without the explicit consideration of interface constraints [23]. Phase field approaches, which were first introduced in the framework of solidification and microstructure development, are encountering ever-expanding applications [24], encompassing fracture mechanics [25,26] or fatigue damage [27,28]. Currently, phase field formulations for environmentally-assisted metal deterioration, such as crack growth aided by hydrogen [29,30] and the progression of the interaction between the liquid electrolyte and the metal due to the dissolution of substances [[31], [32], [33]] have been created. Furthermore, recent research has linked the notions of phase field progression owing to rupture and material dissolution [34]. This paper studied the SCC in the Gulf region in extreme environmental conditions for API X70 steel pipelines under near-neutral ph solution NS4 using Phase Field modelling by using the FRDR mechanism to evaluate the SCC crack and pit propagation for pipelines in oil and gas sectors under various environmental circumstances. The study used material properties and conditions for the oil and Gas pipelines in the Gulf region, and for mimicking this environment, phase field modelling was used and implement the slip dissolution film rupture SCC mechanism and compared with different simulation methods used for the environmental conditions. The simulation was done using the Abaqus user subroutine, and the model was able to capture the crack and pit growth behaviour under different parameters. The modelled was compared with different finite element methods using the strain gradient to determine the SCC crack growth rate under the same environmental conditions.

## 2 SCC under extreme environmental conditions

In some materials, high temperatures and humidity can lead to the rise of SCC. There is indications that excessive humidity can hasten the spread of SCC in some materials, especially when high tensile stress and a corrosive environment are present. For instance, it was discovered that excessive humidity can make stainless steel more susceptible to SCC and that the rate of SCC propagation raised with rising humidity [35]. According to an aviation system analysis, environmental conditions generate 52% of aircraft equipment failure in operation [36], with temperature being the most influential. Simultaneously, when items are used in complicated situations, they are unavoidably influenced by humidity and high temperatures. The examination of meteorological observed data reveals that the temperature in certain regions can reach almost 50° Celsius in the summer. When the high temperature produced by the action of aircraft systems is taken into account, the high temperatures that parts may approach is roughly 60° Celsius. When the relative humidity of the environment exceeds 85%, a nano-thick water coating adheres to the interface of the parts [37]. SCC under Near-neutral pH, caused by diluted soil water that have pH of roughly 7, exhibits a transgranular, quasi-cleavage crack shape including few spreading [38]. Furthermore, near-neutral pH SCC arises over a greater potential range than high pH SCC, that has a restricted width of around 100 mV(SCE) [39]. For further than 2 decades, near-neutral pH SCC has posed an authenticity threat to pipelines in oil and gas sector. Delanty originally reported it in 1985 [40]. Wide area research has revealed that SCC under near-neutral pH conditions is distinguished by broad transgranular cracks [41] and typically arises in dilute near-neutral pH settings with approximately 6.5 pH [42]. A variety of chemical species have been discovered by researchers that produce near-neutral pH SCC. Whereas SCC with high-pH is still not reported, near-neutral pH SCC has arisen in solutions with low concentrations of carbonic acid, bicarbonate ions, as well as other types including chloride, sulphate, and nitrate ions [43]. Through the utilization of intermittent loading experiments, Wang observed that the fracture of X70 steel has the potential to propagate under constant applied loads when exposed to a near-neutral pH surrounding [44]. They also discovered that during continual load testing, SCC under near neutral pH for X70 pipeline steel can occur at an adequate stress and test time [45].

## 3 FRDR mechanism

**Fig. 1:**
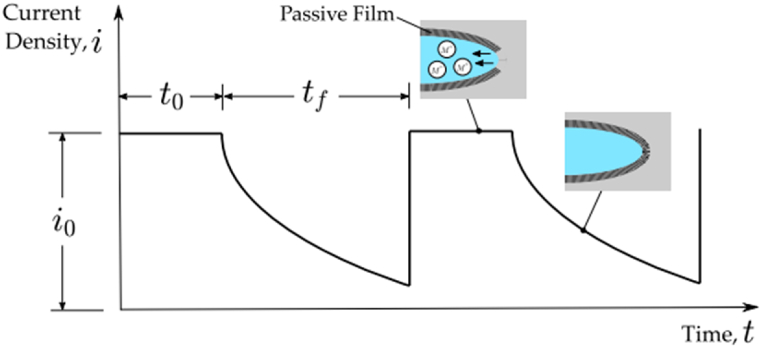
Two cycles of the FRDR process are depicted schematically at the tip of a flaw [31].

The implementation intends to acquire the FRDR processes. When metal alloys are exposed to anodic dissolution conditions, they are protected by a protective layer consisting of metal oxides with a nanometer-scale thickness. This layer effectively acts as a barrier, isolating the material from the corroded medium [46]. Film rupture is an essential requirement for a damaged site in these circumstances, regardless of the particular cracking process. A proposed explanation for pitting corrosion and SCC is the film rupture caused by mechanical straining, preceded by regional metal dissolution [47]. Following every local film rupture incident, repassivation occurs, Under zero-stress conditions, an additional layer is arose on the surfaces of the steel. In a complete cycle regulated with the contest among repassivatin and mechanical loading mechanics, additional mechanical energy is thus required to rupture the new film. As shown in Fig. 1, the improved corrosion resistance rising from the formation of a inactive coating is associated with a reduction in the current density i compared to the current density i_0_ d. A film rupture incident occurred after a fall time t_f_, and the plain metal current density i_0_ is instantaneously regained. This is proceeded by a time interval t_0_ wherein the current flow before decay commences, so the t_i_ = t_0_ + t_f_ indicates a single film rupture-dissolution-repassivation cycle.

According to Ford [48], SCC crack propagation may be described as the method requiring slip dissolution with oxide film rupture. Faraday's Law [49] may be used to define the calculation for the SCC crack propagation rate under slip dissolution conditions:

(1) $$\frac{da}{dt}=\frac{M}{Z.\rho .F}.i\;EQ$$

where da over dt denotes the SCC propagation rate in mm/s. The F-A formula may be stated as follows in Eq. (2) using [50] Eq. (1);

(2) $$\frac{da}{dt}=k_{a}.{\left({\dot{\varepsilon }}_{ct}\right)}^{m}$$

Where k_a_ denotes the oxidative reaction rate, ${\dot{\varepsilon }}_{ct}$ denotes the crack tip strain rate, and m denotes the solution and electrochemistry constants. The material and ambient circumstances close to the crack tip can dictate the oxidative reaction rate constant described by Eq. (3);

(3) $$k_{a}=\frac{M}{Z.\rho .F}\;.\frac{i_{0}}{1-m}\;.\;{\left(\frac{t_{0}}{\varepsilon_{f}}\right)}^{m}$$

Where i_0_ is the current density of the oxidation process, t_0_ is the period prior to the current decays, and $\varepsilon_{f}$ is the strain of the oxidation film rupture. Shoji developed a crack tip strain rate equation in response to the difficulties of measuring the strain rate close to the crack tip when exposed to a constant load state in the F-A equation. According to the crack-tip strain gradient theory in Eq. (4). In the model, the strain rate and macroscopic mechanical properties are coupled [51].

(4) $${\dot{\varepsilon }}_{ct}=\beta \left(\frac{\sigma_{ys}}{E}\right)\left(\frac{N}{N-1}\right)\left(\frac{\dot{a}}{K}\right){\left[\ln\left(\frac{R_{p}}{r_{0}}\right)\right]}^{\frac{1}{N-1}}$$

### 3.1 FRDR phase field model

Using commonly held principles in the literature [52], The mechanical energy required for film formation is quantified by employing an actual plastic strain value, ε^p^, the rupture of the film is expected to transpire when the cumulative actual plastic strain surpasses threshold value, approaching an FRDR period of ε^p^_i_ as shown in Eq. (5):

(5) $$\varepsilon_{i}^{p}=\varepsilon_{f},and\;\varepsilon_{i}^{p}=\int_{0}^{t_{i}}\dot{\varepsilon^{p}dt}$$

where ˙ε^p^ denotes the equivalent plastic strain rate and ε_f_ denotes the film rupture strain threshold, that is in the range of 0.1% [53]. As a result, the period of every FRDR cycle, as well as the value of t_f_, should determined using the corresponding plastic strain's local quantities and duration progression. The decline of the current density is thus defined as an exponential formula of time. Hence, i(t_i_) may be stated throughout a single rupture dissolution repassivation cycle as:

(6) $$i\left(t_{i}\right)=\left\{ \begin{array}{c} i_{0},if\;0<t_{i}\leq t_{0}\\ i_{0}e^{-k\left(t_{i}-t_{0}\right)},if\;t_{0}<t_{i}\leq t_{0}+t_{f} \end{array}\right.$$

where k represents the proneness of corrosion to the durability of the passive film, which is determined by the specific characteristics of the material and the surrounding environment. Mechanical loads and strains are generally limited to the film rupture occurrence. Furthermore, as Gutman showed, residual strains can significantly enhance localized corrosion rates. As a result, improving the term of current density of corrosion using a mechanochemical parameter *k*_m_ [54] as in Eq (7):

(7) $$i_{a}\left(t\right)=\left(\varepsilon_{p},\sigma_{h}\right)i\left(t_{i}\right)k_{m}=\left(\frac{EQ\;\varepsilon_{p}}{\varepsilon_{y}}+1\right)e^{\left(\frac{\sigma_{h}V_{m}}{RT}\right)}i\left(t_{i}\right)$$

where *i*_*a*_ represents the current density associated with electrochemical corrosion, ***ε***_*p*_ represents the equivalent plastic strain, ***ε***_*y*_ represents the yielding strain, ***σ***_ℎ_ represents the hydrostatic stress, *V*_*m*_ represents the molecular volume, T represents the kelvin temperature, and *R* represents the gas constant. Additionally, *k*_m_ is a local term that depends on the local values of ***ε***_*p*_ and ***σ***_ℎ_. Localized corrosion can be driven by either dissolution or fracture processes. The rate at which the boundary of the moving pit advances is described by Faraday's second law in Eq. (8),

(8) $$v_{n}=\frac{i_{a}}{zFc_{s}}$$

Here *F* represents Faraday's constant, *z* represents the charge number and *c*_s_ is the metal atom concentration. Moreover, using the Rankine-Hugoniot criterion, the dissolved ions concentration *c*_*i*_ (x, t) at a location of **x** in the surface may be computed [55] as,

(9) $$\left[D\nabla c_{i}+\left(c_{i}\;\left(x,t\right)-c_{s}\right)v\right].n=0\;EQ$$

The coefficient of diffusion is denoted by D. On the other hand, corrosion is observed to be dissolution controlled when the concentration at the edges of the pit approaches the saturation concentration *c*_sat_. This occurs because of the buildup of metal ions near the pit boundary. The dissipation of metal ions outward from the crack edges governs the velocity of crack advancement. The velocity of the mobile pit edges represented in Eq. (10) can be derived by assuming a saturated concentration in Eq. (9), as follow,

(10) $$v_{n}=\frac{D\nabla c.n}{c_{s}-c_{sat}}$$

**Fig. 2:**
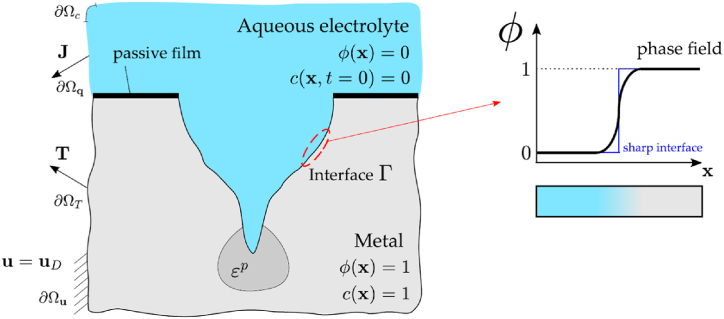
The electrolyte and solid metal phase, and the phase field estimate of the localised corrosion cracking are depicted in the diagram [55].

For the fracture and dissolution controlled operations, simulating the dynamic crack interface necessitates the use of Dirichlet and Robin (*c*_*i*_ equals *c*_sat_) boundary conditions. Alternatively, we avoid these complexities by using the phase field approach described by Ref. [33], indirectly approaching the interface development by computing for a supplementary variable *φ*. The phase field, as demonstrated in Fig. 2, consider amount of 1 for the metal and 0 for the electrolyte, changing uniformly among these two numbers around the interface.

A normalized concentration can be stated as *c* equals *c*_*i*_ over *c*_s_ [33]. Therefore, the normalized concentration *c* is equal to 1 in the metal segment and it tends 0 as the metal-electrolyte interface is approached. The interface kinetics coefficient L, instead of the current density i_a_ governs material dissolution. L value can be conceived of as a constant positive value. In Ref. [55] the authors introduce the concept of a time-related interface kinetics coefficient L to expand the modeling capabilities. They establish a connection between the analysis of the FRDR mechanism and the concept of a mechanochemical increased current density (ia) at a specific time t_i_. Therefore, Eqs. (6), (7) By considering a linear relationship between i_a_ and L, the interface kinetics coefficient during the time period t_i_ can be expressed in Eq. (11);

(11) $$L=\left\{ \begin{array}{c} {\left(\varepsilon_{p},\sigma_{h}\right)k_{m}L}_{0},if\;0<t_{i}\leq t_{0}\\ k_{m}\left(\varepsilon_{p},\sigma_{h}\right)L_{0}e^{-k\left(t_{i}-t_{0}\right)},if\;t_{0}<t_{i}\leq t_{0}+t_{f} \end{array}\right.$$

Martínez-Pañeda (2021) has numerically implemented the PFM to solve the coupled electro-chemical-mechanical problem, where each element contains four degrees of freedoms; u which have 2 components u_x_ and u_y_, *φ*, and c. These degrees of freedom are utilized by three governing equations, The mechanical force balance is expressed in its strongest form [55] as in Eq. (12);

(12) $$\nabla .\left[l\left(\varphi \right)+\kappa )\sigma_{0}\;EQ\right]\;EQ$$

Where ***κ*** represents a minor positive constant presented to avoid entire energy degradation and to maintain the algebraic condition value decently depicted, ***σ***_0_ is the undissolved solid's Cauchy stress tensor, and *l*(*&varphi*) is the formula of decay classifying the transformation from the non-dissolved metal to the phase of electrolyte. The formula of *l*(*&varphi*) should first meet the criteria that *l*(0) equals 0 and *l* (1) equals 1. The phase field balance is the second governing equation [55] and its expressed in Eq (13);

(13) $$\frac{1}{L}\frac{d\varphi }{dt}-2A\left[c-l\left(\varphi \right)\left(c_{Se}-c_{Le}\right)-c_{Le}\right]\left(c_{Se}-c_{Le}\right)l\left(\varphi \right)+wg\left(\varphi \right)-\alpha \nabla^{2}\varphi =0\;EQ$$

Where A represents the free energy density curvature, *c*_Se_ equals to *c*_s_ over *c*_s_ equals to 1 and *c*_Le_ equals to *c*_sat_ over *c*_s_ are the standardized equilibrium concentrations for both phases solid and liquid. the parameters ***α*** and *w* determine the energy ***γ*** and thickness *l* of interface in Eq. (14);

(14) $$w=\frac{6\gamma a^{*}}{l},and,\alpha =\frac{3\gamma l}{a^{*}}\;EQ$$

Where *a*∗ equals 2.94 which is a constant number related to the interface area specification. Experiments may be used to measure the interface energy γ and thickness *l* [56]. Following on to the mass balance equation is written in Eq. (15),

(15) $$\frac{dc}{dt}-\nabla .D\nabla \left(c-l\left(\varphi \right)\left(c_{Se}-c_{Le}\right)-c_{Le}\right)=0$$

This is an expansion of Fick's second process in which metal ion diffusion occurs exclusively along the surface and in the electrolyte. Emilio uses a user element subroutine to build the finite element model using ABAQUS UEL user subroutine.

## 4 Material

API 5L X70 and X60 steel are utilized in the oil and gas pipeline industry within the GCC petroleum sector due to their favorable combination of high strength-to-weight ratio, fracture toughness, and, importantly, yield strength [57]. In all the electrochemistry studies conducted, a corrosive environment resembling soil solution (NS4) with a pH close to neutral was used. To simulate the near-neutral pH stress corrosion cracking (SCC) response, researchers commonly employ a synthetic solution known as NS4, which replicates soil conditions. Other studies have also utilized various simulated soil solutions such as NS1, NS2, NS3, and NS4 [58]. Table 1 displays the chemical structure of the NS4 solution used in this analysis.

**Table 1:** NS4 solution Chemical composition (g/L) [58].

KCI	NaHCO_3_	MgSO_4_.7H_2_O	CaCl_2_. 2H_2_O
0.122	0.483	0.131	0.181

In NS4 solution, X70 steel is susceptible to corrosion due to the incidence of chloride ions, which can initiate pitting and crevice corrosion. Several factors, including the concentration of chloride ions, temperature, pH, and the presence of other ions or contaminants can influence the corrosion behaviour of X70 steel in NS4 solution [59]. In soil solution (NS4), the elastic properties of API X70 steel will be affected by several elements, such as the temperature and humidity of the soil, the chemical composition of the soil, and the thickness and diameter of the steel pipe. Table 2 shows the elastic properties of API X70 steel when exposed to a soil solution (NS4) [60].

**Table 2:** Elastic properties of X70 steel under NS4 [60].

Material Name	Elastic Modulus (GPa)	Poisson's Ratio	Yield Stress (MPa)	Density (kg/m^3^)
API 5l X70	205	0.28	600	7850

## 5 Finite element simulation Setup

**Fig. 3:**
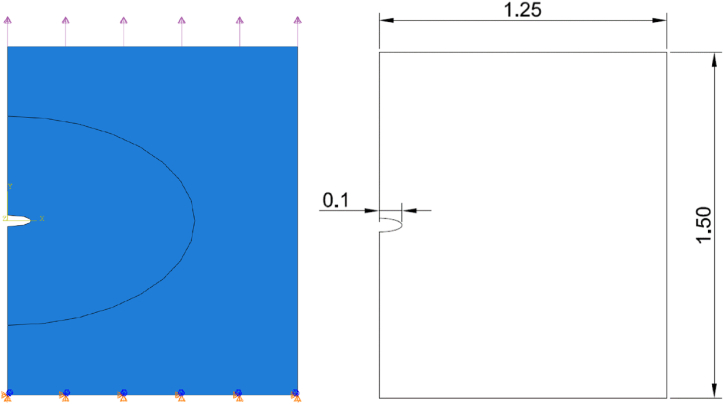
geometry model and boundary conditions.

The simulation was done using Abaqus CAE software [61]. A user element user-subroutine in ABAQUS is utilized to create the numerical model (UEL). The use of user subroutine to implement the phase field modelling for FRDR mechanism. The subroutine is mainly done by Emilio [55]. The UEL subroutine is employed to solve the governing equations for displacement, phase field, and concentration within this model. The simulation was done using coupled temp-displacement analysis, and the initial pit was identified as a crack. A plane strain plate shown in Fig. 3 with an area of 1.25 mm by 1.5 mm with an initial 0.1 mm crack pit depth, fixed boundary condition assumed at the bottom boundary. An applied tensile constant loading on the top boundary of 150 MPa. The boundary conditions *φ* = 0 and *c* = 0 are referred to as Dirichlet boundary conditions and are applied at the surface of the initial pit. The initial pit has a arched shape with radii of 0.1 mm. At the plate, the initial conditions are specified as *t* = 0, *c* = 1, and *φ* = 1. The mechanical properties of the solid are decided by its ability to deform elastically and plastically under applied forces or stresses. It also incorporates work hardening, which describes how materials become harder when they experience repeated loading cycles over time due to plastic deformation caused by external loads. Furthermore, the mechanical properties of a solid are affected by film passivation, which is the protective effect of thin oxide films on metal surfaces that prevent corrosion, and by local corrosion phenomena, for instance pitting and stress corrosion cracking. These forms of corrosion can be driven by both electrochemical and mechanical factors.

Following [30,33], we evaluate the material and corrosion characteristics presented in Table 3; the corrosion kinetics is utilized by a steady-state parameter *L*, which is equal to *L*_0_. If L_0_ is high, the mechnism is dissolution-controlled, meaning that the electrochemical process at the metal-electrolyte interface occurs rapidly and results in the attainment of a maximum or saturated surface concentration of metal ions. Alternatively, if *L*_0_ is low, the corrosion process is fracture-controlled, and the crack length shows a linear relation with time. the quantities mentioned for the thickness of the interface *l* and its energy ***γ*** derive from the options of a gradient energy coefficient ***α*** equals 1 × 10−3 N and a double well potential height *w* equals 10 N/mm2.

**Table 3:** Electrochemical and phase field parameters [33].

Parameter	Value	Unite
Diffusion Coefficient	8.5e-4	mm2/s
Interface energy	0.02	N/mm
Interface thickness	0.04	mm
Curvature of the Free energy density, A	53.5	N/mm2
Interface kinetics coefficient, L0	1e-4	mm2/(N.s)
Film Rupture strain	0.003	–
Time period prior to new passive film formation	50	s

**Fig. 4:**
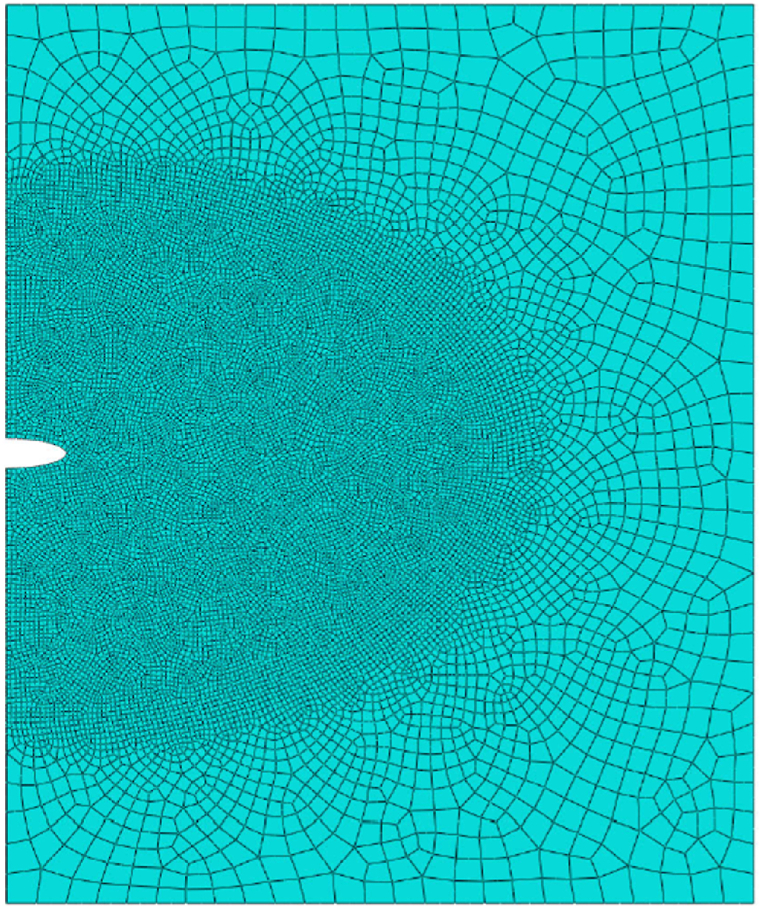
Mesh.

A consistent mesh is applied, with an element size about 15 times less than the interface thickness and utilizing a number of 14,857 plane strain quadratic 2d elements with a refinement around the crack pit to capture the crack growth profile precisely as illustrated in Fig. 4. The model simulations are implemented at the microscale, and the range of applicability of the model is limited to elastic-plastic solids.

The simulation will study the effect of different corrosion parameters by changing the Film Rupture strain, Interface kinetics coefficient, and k. then the model will be compared with other film rupture slip dissolution models.

## 6 Validation

### 6.1 SCC comparative study

**Fig. 5:**
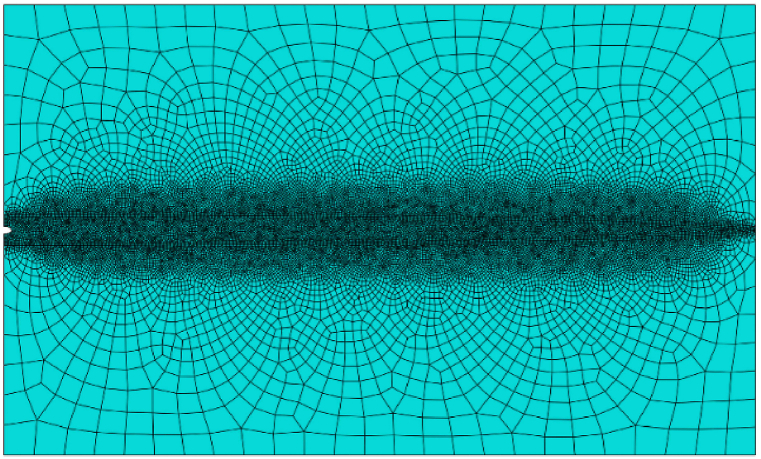
Geometry and Mesh for the comparative model.

This comparative study aims to verify our model under a near-neutral NS4 solution for X70 steel by comparing the results with another numerical method for crack propagation in Ref. [62] using the same material and environmental conditions. The study uses the j-integral theory in COMSOL multi-physics, and the crack growth rate is determined using the strain gradient method obtained numerically. Fig. 5 shows the geometry and the mesh, the width is 0.5 mm, the height is 0.3 mm, and the semi-elliptical tip is 0.005 mm. the mesh has 24779 quadrilateral quadratic element and 74462 of nodes, and the refinement was done in the area of the propagation. The boundary conditions are the same as that used in the parametric study with constant loading of 200 MPa, the same boundary conditions were applied in the comparison study.

The boundary conditions are the same as that used in the parametric study with constant loading of 200 MPa, the same boundary conditions were applied in the comparison study. Table 4 shows the phase field and electrochemistry parameters, which are the same used in Ref. [62] and will be used for verification. L_0_ is selected as the initial interface kinetics coefficient to give a suitable match to the numerical analysis results. here, L_0_ equals to 1.2 × 10^−9^ mm^2^ ∕ (N s).

**Table 4:** Electrochemical and phase field parameters for comparative study.

Parameter	Value	Unite
Diffusion Coefficient	5.41e-4	mm2/s
Interface energy	0.1	N/mm
Interface thickness	0.004	mm
Free energy density curvature, A	733	N/mm2
Interface kinetics coefficient, L0	1.2e-9	mm2/(N.s)
Film Rupture strain	0.0025	–
Time interval before new passive film formed	10	s

**Fig. 6:**
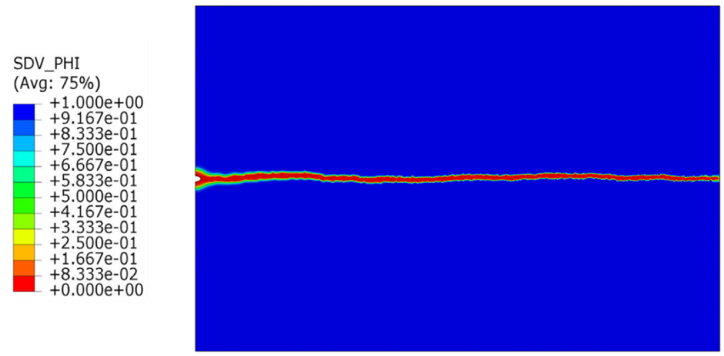
Contours of the phase field.

The material parameter and environmental conditions are discussed under the material section, the same properties are used in both studies. Fig. 6 shows the phase filed contours that represent the crack growth, these contours were obtained after 107 million seconds. The crack propagated as a straight line due to the pure tension constant loading, and the crack was mostly activation controlled based due to the lower value of the mobility parameter L_0_.

**Fig. 7:**
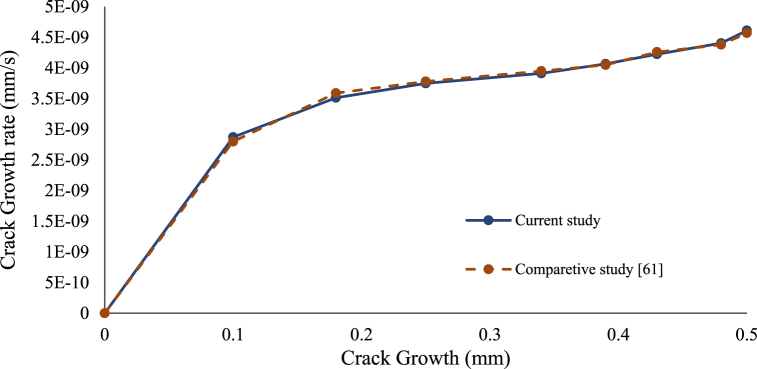
Crack Growth rate against crack length.

The crack propagation rate was obtained to compare the results between the studies for verification. Fig. 7 illustrate the crack propagation rate against the crack size, as the crack propagates and increases the crack propagation rate rise owing to the crack growth rate's correlation to the normal plastic strain which grows with the crack length. The results reveal a decent agreement with the comparative study.

## 7 Results and discussion

### 7.1 SCC parametric study

**Fig. 8:**
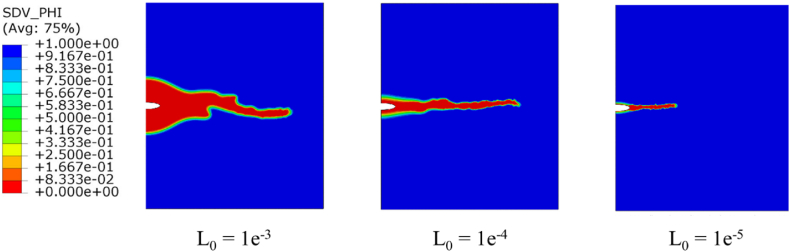
SCC riginating from a semi-elliptical crack contours of the phase field, depicting different values of L_0_.

Numerical tests were conducted on an X70 steel plate comprising a previously formed defect to examine the impact of stresses and repassivation on the progression of SCC. First, we will explore the impact of the mobility parameter L_0_, which has been made dependent on mechanical aspects in order to include two major impacts: (a) the influence of mechanical loading in fracturing the protective oxide layer, and (b) The incorporation of mechanical domains plays a role in enhancing the electrochemical properties. If L_0_ is relatively high, the operation will be considered dissolution driven, with the corrosion reaction at the metal-electrolyte surface arising extremely quickly and resulting in saturation of metal surface concentration. If L_0_ is low, the corrosion method will be considered fracture controlled, and the crack length has a linear relationship with the time. Fig. 8 shows the results for various values of L_0_ equal to 1e-3, 1e-4, and 1e-5. For L_0_ equals to 1e-3 the contour captured at 4200 s, the crack growth started as diffusion-controlled pit growth, so the crack is driven by the corrosion parameter, then the crack growth at 2200 s converted to fracture controlled crack growth so the crack is deriven by the electrochemistry parameters and the mechanical load. As the L_0_ decrease, the crack gets thinner and tended to be activation-based crack growth.

**Fig. 9:**
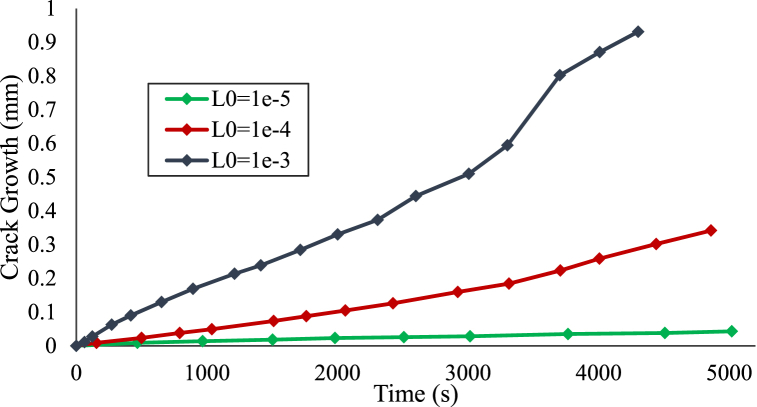
Crack growth with time for different L0 values.

Fig. 9 illustrate the crack growth against the time plot for the three cases. If L_0_ was minimal, the corrosion mechanism is fracture controlled, and the size of the crack exhibits a Directly proportional to time. Although it is obvious that raising the interfacial reaction rate constant rises the size of the crack length, this responsiveness to L_0_ is non-linear and dissolves as L_0_ grows; for a reasonably enormous amount of L_0_, rising its value does not influence the kinetics of corrosion processes. Corrosion turns into diffusion-limited at this point, and a greater amount of D would be used to boost dissolving rates. As the L0 raises the crack growth increases with time, for L0 equals 1e-4 and 1e-5 the crack is raising linearly. However, for L0 equals 1e-3 the increase started linearly till 2400 s, and then the crack growth behaviour started to be nonlinear. Moreover, When the mechanical amplification of L exceeds a certain threshold, it leads to the attainment of dissolution-controlled behavior, resulting in saturation of the mechanical impact. Thus, mechanical domains have the greatest impact on activation-controlled corrosion.

**Fig. 10:**
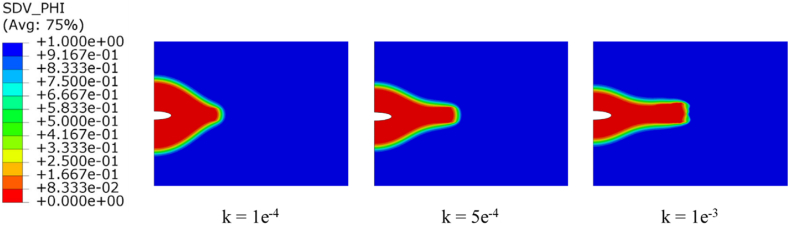
Contours of the phase field with different k values.

Turning into the repassivation process and FRDR formulation. Therefore, a constant loading of 150 MPa is applied, and the susceptibility of the results is investigated in relation to variations in the material property that describes the corrosion susceptibility to the resilience of the oxide layer. There are three typical values considered for k which are 1 × 10^−4^, 5 × 10^−4^, and 1 × 10^−3^. Fig. 10 illustrates the phase field contours observed at the end of a 2000-s period. High values of the parameter k result in an accelerated rate of SCC propagation at the center of the pit compared to the pit mouth. This process leads to the refinement of the pit and its transformation into a crack. As the crack refines and plasticity becomes localized, corrosion rates increase towards the crack tip. It is noteworthy that the shielding coating is insufficient in areas experiencing significant mechanical stress.

**Fig. 11:**
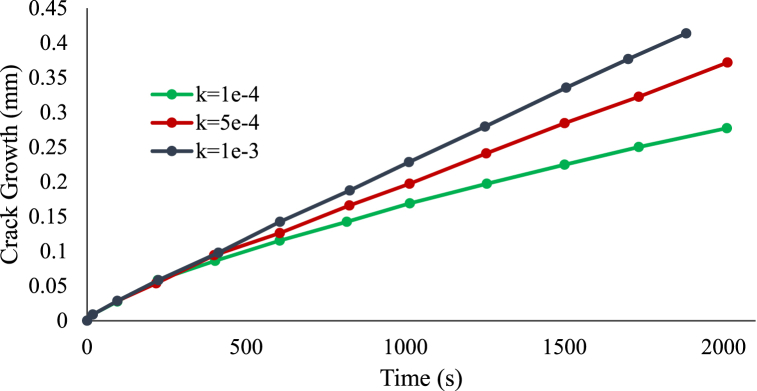
Crack growth with time for different k values.

Fig. 11 depicts the length of the SCC area with respect to the film stability parameter k. The results show that when the repassivation operation is introduced, the size of the SCC area decreases slightly at a preliminary phase, which is consistent with the notion that repassivation restrains the SCC operation by reducing the amount of the surface reaction rate coefficient. Following a given period, when the film ruptures, the size of the SCC area expands quicker with rising k, and the amount of the corresponding plastic strain rises as the crack refines, amplifying the surface reaction rate coefficient. The maximum crack growth was 0.4 mm for k equal to 1e-3, and the minimum was 0.26 mm for k equal to 1e-4.

**Fig. 12:**
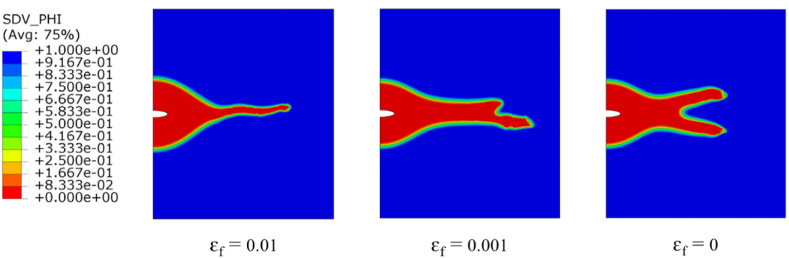
Contours of the phase field with different ***ε***_f_ values.

We conclude our parametric study by analyzing how anodic dissolution influences resistance to film breakage. Fig. 12 shows the crack growth behaviour for different film rupture strain values at 4700 s, the crack growth for the three cases is around 0.4 mm, however, the difference between them is the crack growth behaviour. For higher values of film rupture strain, the growth was pit growth (diffusion controlled) for a longer time than the lower film rupture strain values, then it's converted to activation controlled based crack growth. For zero film rupture strain after 3000 s, the crack bifurcated into two directions which considered that there is no film rupture occurs on the crack tip.

## 8 Conclusion

The SCC crack propagation and pit growth under near-neutral environmental conditions were analyzed using phase field modelling. X70 steel under NS4 the solution was used for the analysis to represent the slip dissolution film rupture mechanism. A parametric study was done to study the impact of different electrochemistry and phase field parameters on crack growth behaviour. Under varied circumstances, SCC from an spheroidal pit is anticipated to analyze the effect of corrosion parameters and their interaction with the FRDR mechanism. The corrosion rates are influenced by the interface kinetics coefficient L. As L increases, corrosion rates also increase, transitioning from fracture-controlled to dissolution-controlled corrosion. However, once the system reaches a dissolution-restricted state, further increases in L do not significantly affect corrosion rates. High values of the parameter k result in an accelerated rate of SCC propagation at the base of the crack compared to corrosion rates at the crack mouth. This process leads to the refinement of the pit and its transformation into a crack. Comparison research was conducted to validate our model under a near-neutral NS4 solution for X70 steel, with the results compared to other numerical methods for crack propagation utilizing the same material and environmental conditions. The results reveal a decent agreement with the comparative study. Future advancements might include expanding the model to include cracking processes caused by cathodic reactions such as hydrogen embrittlement.

## Funding

The research conducted in this study is funded by the ASPIRE Award for Research Excellence (AARE 2019) through Project Number AARE19-098, provided by the Advanced Technology Research Council-ASPIRE.

## Author contribution statement

Abdullah Alsit: Conceived and designed the experiments; Wrote the paper. Hasan Hamdan: Performed the experiments. Aghyad B. Al Tahhan: Contributed reagents, materials, analysis tools or data. Mohammad Alkhedher: Analyzed and interpreted the data.

## Data availability statement

Data will be made available on request.

## Declaration of competing interest

The authors declare that they have no known competing financial interests or personal relationships that could have appeared to influence the work reported in this paper.
